# Gut Microbiome Changes Associated With HIV Infection and Sexual Orientation

**DOI:** 10.3389/fcimb.2020.00434

**Published:** 2020-09-24

**Authors:** Jie Zhou, Yu Zhang, Ping Cui, Lijia Luo, Hui Chen, Bingyu Liang, Junjun Jiang, Chuanyi Ning, Li Tian, Xiaodan Zhong, Li Ye, Hao Liang, Jiegang Huang

**Affiliations:** ^1^Guangxi Key Laboratory of AIDS Prevention and Treatment, Guangxi Universities Key Laboratory of Prevention and Control of Highly Prevalent Disease, Guangxi Medical University, Nanning, China; ^2^School of Public Health, Guangxi Medical University, Nanning, China; ^3^Guangxi Collaborative Innovation Center for Biomedicine, Life Science Institute, Guangxi Medical University, Nanning, China; ^4^The First Affiliated Hospital of Guangxi Medical University, Nanning, China

**Keywords:** HIV, AIDS, sexual orientation, gut microbiome, 16S rRNA gene amplicon sequencing

## Abstract

**Background:** Many studies have explored changes in the gut microbiome associated with HIV infection, but the consistent pattern of changes has not been clarified. Men who have sex with men (MSM) are very likely to be an independent influencing factor of the gut microbiome, but relevant research is still lacking.

**Methods:** We conducted a meta-analysis by screening 12 published studies of 16S rRNA gene amplicon sequencing of gut microbiomes related to HIV/AIDS (six of these studies contain data that is relevant and available to MSM) from NCBI and EBI databases. The analysis of gut microbiomes related to HIV infection status and MSM status included 1,288 samples (HIV-positive (HIV+) individuals, *n* = 744; HIV-negative (HIV–) individuals, *n* = 544) and 632 samples (MSM, *n* = 328; non-MSM, *n* = 304), respectively. The alpha diversity indexes, beta diversity indexes, differentially enriched genera, differentially enriched species, and differentially enriched Kyoto Encyclopedia of Genes and Genomes (KEGG) functional pathways related to gut microbiomes were calculated. Finally, the overall trend of the above indicators was evaluated.

**Results:** Our results indicate that HIV+ status is associated with decreased alpha diversity of the gut microbiome. MSM status is an important factor that affects the study of HIV-related gut microbiomes; that is, MSM are associated with alpha diversity changes in the gut microbiome regardless of HIV infection, and the changes in the gut microbiome composition of MSM are more significant than those of HIV+ individuals. A consistent change in *Bacteroides caccae, Bacteroides ovatus, Bacteroides uniformis*, and *Prevotella stercorea* was found in HIV+ individuals and MSM. The differential expression of the gut microbiome may be accompanied by changes in functional pathways of carbohydrate metabolism, amino acid metabolism, and lipid Metabolism.

**Conclusions:** This study shows that the changes in the gut microbiome are related to HIV and MSM status. Importantly, MSM status may have a far greater impact on the gut microbiome than HIV status.

## Introduction

Early studies have shown that the intestinal mucosa is the primary site of early HIV-1 reproduction, irrespective of the way in which HIV-1 invades the body, whether by sexual contact or blood transfusion (Mehandru et al., 2004). HIV-1, which enters the intestinal mucosa at the very early stages of infection, can cause the Th17 CD4^+^T cells of the intestine to be destroyed and depleted and the integrity of the intestinal mucosa to be impaired (Epple et al., 2010; Hirao et al., 2014). In addition, gut microbiome translocation can occur, and the gut microbiome and its products can enter the systemic blood circulation (Balagopal et al., 2008), eventually leading to activation of the immune system and spread of the HIV-1 infection (Brenchley et al., 2006).

In recent years, exploration of the role and mechanism of the gut microbiome in the development of HIV infection has gradually become a popular topic of academic research. However, there is still inconsistent evidence about the alpha diversity and composition of the gut microbiome after HIV infection. Most current studies suggest that HIV+ status is related to the downregulation of alpha diversity in the gut microbiome (Mutlu et al., 2014; Yu et al., 2014; Nowak et al., 2015; Dubourg et al., 2016; Noguera-Julian et al., 2016; Pinto-Cardoso et al., 2017; Vesterbacka et al., 2017; Villanueva-Millan et al., 2017). Some researchers (McHardy et al., 2013; Dinh et al., 2015; Nowak et al., 2017) also compared the alpha diversity of the gut microbiome in HIV+ and HIV– individuals, but no significant difference was found. The study by Lozupone et al. (2013) showed that the alpha diversity of the gut microbiome in HIV+ individuals who did not receive antiretroviral therapy (ART) was significantly higher than that of HIV– individuals. Moreover, in many studies, there are inconsistent results regarding the change in the composition of the gut microbiome after HIV infection. Some studies have shown that the abundance of *Prevotella* increases significantly and the abundance of *Bacteroides* decreases significantly in HIV+ individuals compared to HIV– individuals (Vujkovic-Cvijin et al., 2013; Dillon et al., 2014; Mutlu et al., 2014; Vázquez-Castellanos et al., 2015; Sun et al., 2016; Yang et al., 2016; Armstrong et al., 2018; Neff et al., 2018). However, a study by Noguera-Julian et al. (2016) showed that the increase in the *Prevotella*/*Bacteroides* ratio is associated with MSM status rather than HIV status, which has since been corroborated by several other studies (Armstrong et al., 2018; Neff et al., 2018; Li et al., 2019). Although many studies have explored the changes in the gut microbiome associated with HIV infection, the pattern of these changes has not been elucidated. MSM status is very likely an independent influencing factor of the gut microbiome, but there is still a lack of relevant research to explore it.

In addition, HIV infection can cause dysregulation of multiple functional pathways in the human body (Vázquez-Castellanos et al., 2015, 2018). On the one hand, HIV-related gut microbiomes are well-adapted to inflammatory environments, such as the high expression of the anti-oxidative stress response pathway and the low expression of the anti-inflammatory response process. On the other hand, the gut microbiome can promote the occurrence and development of intestinal inflammation. Therefore, exploration of the functional changes related to HIV infection based on the gene expression profile of the gut microbiome can increase our understanding of the interaction between the gut microbiome and the human body.

To clarify the diversity of the gut microbiome related to HIV infection, to determine whether MSM status is an independent factor influencing the gut microbiome, and to explore the consistent change in the gut microbiome and functional pathways in HIV+ individuals and MSM, we screened 12 published studies of 16S rRNA gene amplicon sequencing of the gut microbiome related to HIV/AIDS (six of these studies contain data that is relevant and available to MSM) from NCBI and EBI databases. The alpha diversity indexes, beta diversity indexes, genera, species, and KEGG functional pathways related to the gut microbiome were calculated. Finally, the overall trend in the above indicators was evaluated.

## Materials and Methods

### Research Strategy

**Figure 1:**
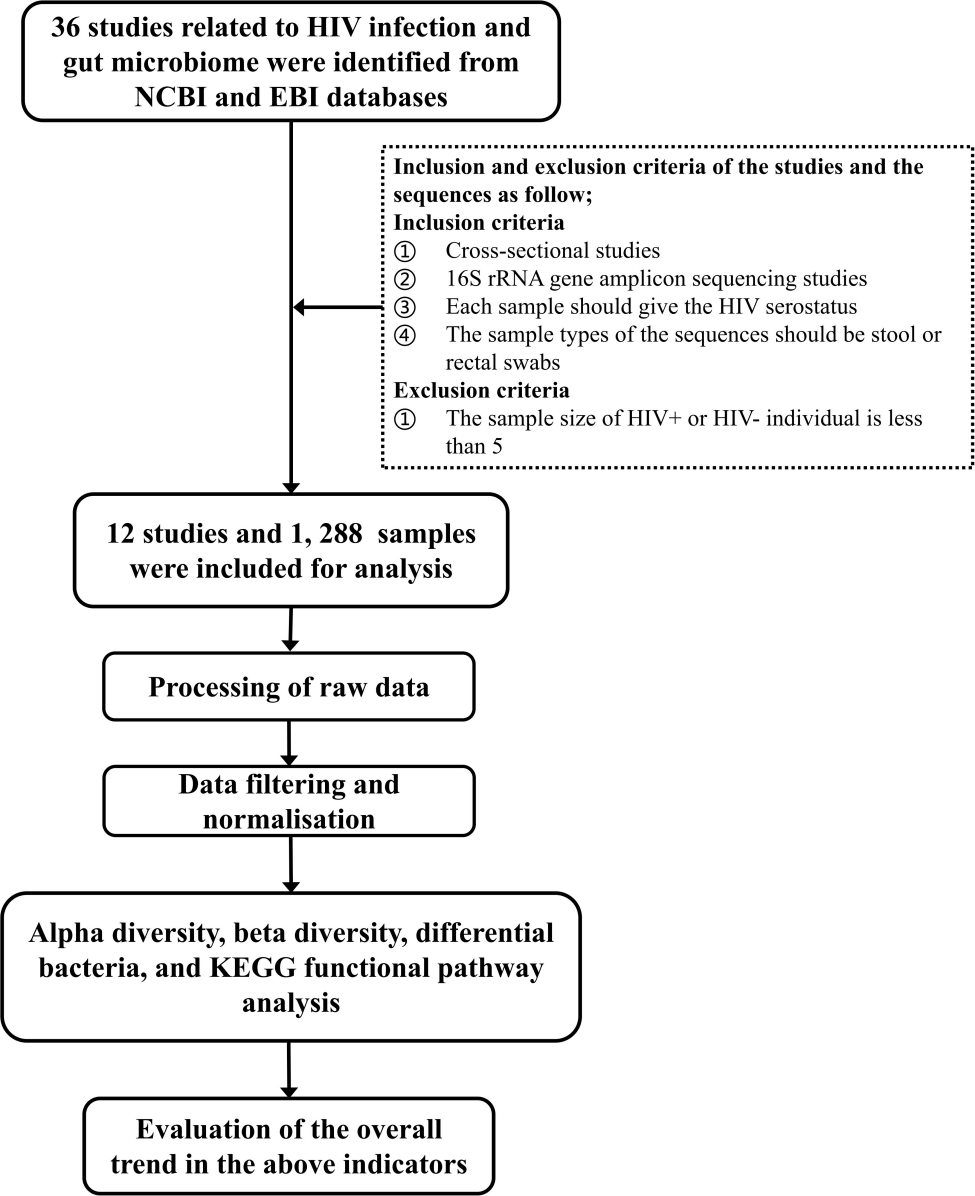
The technical route of the study.

Studies of human fecal flora related to HIV/AIDS by 16S rRNA gene amplicon sequencing before October 2019 were retrieved from the NCBI and EBI databases. Studies were screened according to the following inclusion and exclusion criteria: (1) cross-sectional studies, (2) each sample should give the HIV status of the corresponding subject, (3) the sample types of the sequences should be stool or rectal swabs, and (4) the sequencing method should be 16S rRNA gene amplicon sequencing. Studies with sample sizes of HIV+ or HIV– individuals of <5 were excluded. The technical route of the study is shown in Figure 1.

### Processing of Raw Data

The raw sequences were processed using QIIME version 1.9 (Caporaso et al., 2010), and the general process included FLASH software (Magoc and Salzberg, 2011) used for splicing paired-end 16S rRNA gene reads. After splicing, Cutadapt was used for removing primers from the sequences, and low-quality sequences were removed. Based on the chimera database of UCHIME (Edgar et al., 2011), Usearch version 6.1.554 was used to identify and remove chimeras in the sequences, and open reference operational taxonomic unit (OTU) picking was performed with UCLUST (Edgar, 2010) against the Greengenes database (DeSantis et al., 2006), version 13.8, with a similarity of at least 97% (Rideout et al., 2014). The analysis after clustering used the platforms of MicrobiomeAnalyst (Dhariwal et al., 2017), R 3.5.1, Galaxy (Goecks et al., 2013), and REVMAN 5.3.

### Data Filtering

Data filtering is done to remove low quality or uninformative features to improve downstream statistical analysis. The minimum count and prevalence in the samples (%) were filtered according to the characteristics of each dataset, which may be caused by sequencing errors or low levels of contamination in the sample. At the same time, for the features that were close to constant throughout the experiment, which are conditions that are not likely to be associated with the conditions under study, we used the interquartile range (IQR) to detect their variances and filters.

### Data Normalization

To address the variability in the sampling depth and the sparsity of the data in order to enable more biologically meaningful comparisons, we used total sum scaling (TSS) to bring all samples to the same scale by dividing the samples by a scaling factor.

### Data Analysis

At the OTU level, to assess alpha diversity, richness (Observed, Chao1, and ACE) and diversity (Shannon, Simpson, Fisher, and Invsimpson) indexes were calculated. Differences between two groups were analyzed via the Student's *t*-test and the Mann–Whitney *U*-test. For principal coordinates analysis (PCoA), distance matrices were calculated using the Bray–Curtis, Jensen–Shannon divergence, and Jaccard ecological dissimilarity indexes. The permutational multivariate analysis of variance (PERMANOVA) test was performed on this distance matrix.

The function of the gut microbiome was inferred using a phylogenetic investigation of communities by reconstruction of unobserved states (PICRUSt) (Langille et al., 2013) in the Greengenes database. In brief, the general process corrected the OTU table for multiple 16S copy numbers. Then, the normalized phylotype abundance was multiplied by the respective set of gene abundances, represented by the KEGG, to identify estimates for each taxon. The accuracy of the KEGG prediction results was evaluated by the nearest sequenced taxon index (NSTI). For the identification of different genera, species, and KEGG functional pathways, we used the linear discriminant analysis effect size (LEfSe) method to perform the identification (LDA score was ≥2) and the DESeq2 and Random Forests methods to verify the results of LEfSe.

REVMAN 5.3 software was used to build the forest plots based on the alpha diversity indexes, and *Chi*^2^ and *I*^2^ were used for heterogeneity testing for each study. If *p* > 0.1 or *I*^2^ < 50%, the constructed model is not heterogeneous. Sensitivity analysis removes the study with the largest sample size and non-European/non-U.S. studies and converts the fixed effects model (FEM) to the random effects model (REM). All *p*-values were corrected for multiple comparisons through the false discovery rate (FDR) technique. All tests were two-sided, and an FDR *p* < 0.05 was considered statistically significant.

## Results

### Study Description

A total of 36 studies related to HIV infection were retrieved from the NCBI and EBI databases. Twelve studies were finally selected for subsequent analysis. The total number of samples included in the overall analysis was 1,288 (HIV+ individuals, *n* = 744; HIV– individuals, *n* = 544). Six of the 12 studies contain data that is relevant and available to MSM status, including 632 samples (MSM, *n* = 328; non-MSM, *n* = 304). The metadata variables were HIV status, age, gender, body mass index (BMI), MSM status, ART use, CD4^+^T cell count, and HIV viral load (VL). A summary of the included studies is presented in Table 1. The unfiltered sequencing quality results are presented in Supplementary Table 1.

**Table 1:** Summary of the included studies.

**References**	**Title**	**Bioproject accession number**	**Sample size**		**Age**		**CD4^**+**^T cell count**	**HIV viral load**	**Man/Woman**		**MSM/non-MSM**		**Treatment**	**16S rRNA variable region/Sequencing platform**	**Country**
			**HIV+**	**HIV-**	**HIV+**	**HIV-**			**HIV+**	**HIV-**	**HIV+**	**HIV-**			
Lozupone et al. (2013)	Alterations in the Gut Microbiota Associated with HIV-1 Infection	PRJEB4335	30	22	29 (34.7) 1 unknown	22 (37.5)	30 (584.9)	30 (45594.6)	Unknown	Unknown	Unknown		16 ART users14 non-ART users	V4/ Illumina Miseq	USA
Dillon et al. (2014)	An altered intestinal mucosal microbiome in HIV-1 infection is associated with mucosal and systemic immune activation and endotoxemia	PRJNA227062	18	14	18 (32.5)^*^	14 (31)	18 (425)	18 (51350)	13/5	9/5	Unknown		18 non-ART users	V4/Illumina Miseq	USA
Dinh et al. (2015)	Intestinal Microbiota, Microbial Translocation, and Systemic Inflammation in Chronic HIV Infection	PRJNA233597	21	15	21 (50.2)	15 (44.0)	21 (741)	19 (574) 2 unknown	17/4	11/4	12/4	0/4	21 ART users	V3-V5/Roche GS FLX	USA
Vázquez-Castellanos et al. (2015)	Altered metabolism of gut microbiota contributes to chronic immune activation in HIV+ individuals	PRJEB5185	9	12	Unknown		Unknown	Unknown	Unknown	Unknown	Unknown		9 ART users	V1, V2, and V3/Roche GS FLX	Spain
Dubourg et al. (2016)	Gut microbiota associated with HIV infection is significantly enriched in bacteria tolerant to oxygen	PRJEB10578	56	50	Unknown		Unknown	Unknown	Unknown	Unknown	Unknown		Unknown	V3-V4/Illumina MiSeq	France
Noguera-Julian et al. (2016)	Gut Microbiota Linked to Sexual Preference and HIV Infection	PRJNA307231	206	34	206 (42.8)	34 (40.0)	205 (613) 1 unknown	123 (92848)82 undetectable1 unknown	147/59	29/5	96/110	23/11	58 non-ART users 71 ART users 77 unknown	V3-V4/Illumina MiSeq	Spain/ Sweden
Vesterbacka et al. (2017)	Richer gut microbiota with distinct metabolic profile in HIV infected Elite Controllers	PRJNA354863	47	15	47 (46)	15 (49.9)	Unknown	Unknown	24/23	7/8	12/34 1 unknown	3/12	15 ART users 32 non-ART users	V3-V4/Illumina MiSeq	Sweden
Armstrong et al. (2018)	An exploration of *Prevotella*-rich microbiomes in HIV and men who have sex with men	PRJEB28485	112	105	112 (43.6)	105 (34.8)	112 (674)	112 (58713)	92/19 1 FTM	64/41	90/22	35/70	67 ART users 45 non-ART users	V4/Illumina MiSeq	USA
Cook et al. (2019)	Effects of HIV Viremia on the Gastrointestinal Microbiome of Young Men who have Sex with Men	PRJNA422134	183	200	383 (31)		183 (625)	Unknown	383 male		383 MSM		Unknown	V4/Illumina MiSeq	USA
Lee et al. (2018)	Enrichment of gut-derived *Fusobacterium* is associated with suboptimal immune recovery in HIV+ individuals	PRJNA489590	26	20	26 (42.6)	20 (37.2)	26 (639)	Undetectable	26 male	20 male	14/12	11/9	26 ART users	V4/Illumina MiSeq	Malaysia
Neff et al. (2018)	Fecal Microbiota Composition Drives Immune Activation in HIV+ individuals	PRJEB25418	24	21	24 (46.6)	21 (37.7)	24 (593)	23 (57654)1 unknown	17/7	16/5	Unknown		15 ART users9 non-ART users	V2/Illumina MiSeq	USA
Li et al. (2019)	Gut microbiota from high-risk men who have sex with men drive immune activation in gnotobiotic mice and *in vitro* HIV infection	PRJEB31328	12	36	Unknown		Unknown	Unknown	48/0	NA	12/0	20/16	12 non-ART users	V2/Illumina MiSeq	USA

*MSM, men who have sex with men; ART users, HIV+ ART (antiretroviral therapy) users received suppressive ART for > 12 months; non-ART users, HIV+ non-ART users were no ART exposure / <10 days of ART at any time prior to entry / not been on treatment for 47 days in the preceding 6 months / at least 6 months in the absence of ART; FTM, Female to male transgender*.

* *Sample size (Mean)*.

### Richness and Diversity of the Gut Microbiome Based on HIV Status

**Figure 2:**
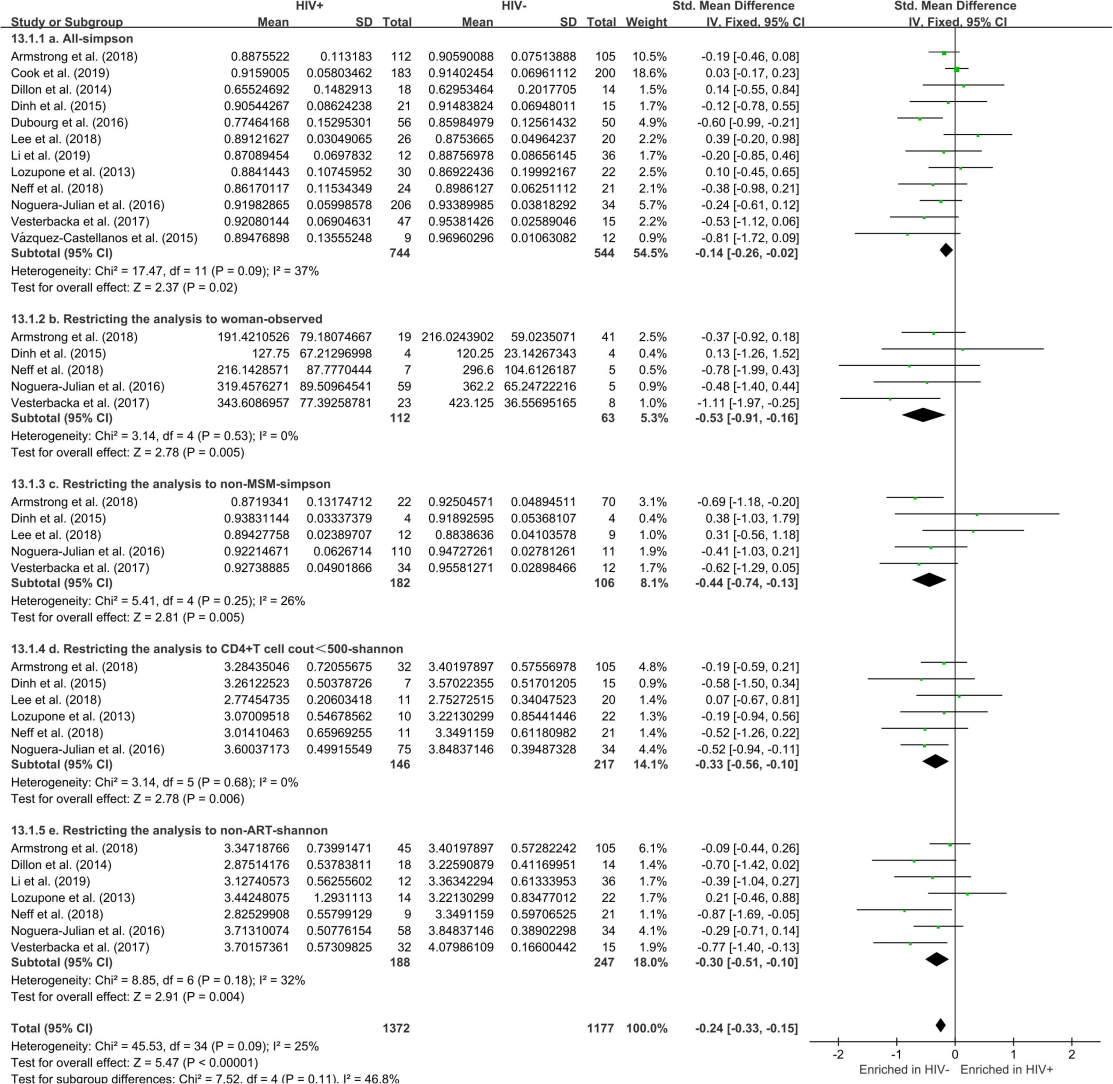
Forest plots comparing HIV+ to HIV– individuals. The fixed effects models (FEMs) with a 95% CI above or below zero were considered statistically significant. The heterogeneity analysis included estimates of *Chi*^2^ and *I*^2^. Before controlling for other confounding factors, the alpha diversity of the HIV+ individuals was significantly lower than that of the HIV– individuals (**a**, Simpson index). When restricting the analysis to women (**b**, Observed index), non-MSM individuals (**c**, Simpson index), individuals with CD4^+^T cell count of <500 (**d**, Shannon index), and non-ART individuals (**e**, Shannon index), HIV+ status was associated with a significant decrease in alpha diversity.

**Figure 3:**
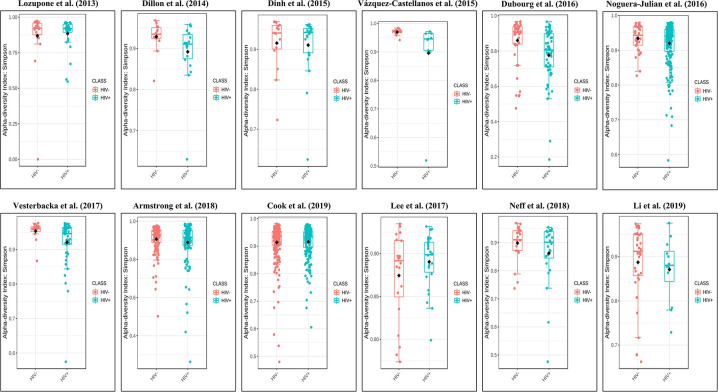
Boxplots showing the alpha diversity in terms of the Simpson index by study and HIV status (red, HIV– individuals; blue, HIV+ individuals). Most studies showed decreased alpha diversity in HIV+ individuals compared to HIV– individuals.

The calculation results of the alpha diversity indexes of the OTU level of 12 studies are shown in Table 2. Before controlling for other confounding factors, the alpha diversity of the HIV+ individuals was significantly lower than that of the HIV– individuals, including the ACE (*Z* = 2.92, FDR *p* = 0.009), Shannon (*Z* = 3.44, FDR *p* = 0.004), Simpson (*Z* = 2.37, FDR *p* = 0.028), and Insimpson indexes (*Z* = 2.85, FDR *p* = 0.009) of the FEM. The results after the sensitivity analysis remained consistent. The forest maps (Figure 2a, Supplementary Figure 1) and boxplots (Figure 3) of the 12 studies included also showed this trend.

**Table 2:** The alpha diversity results of HIV in OTU level.

**References**	**Observed**	**Chao1**	**ACE**	**Shannon**	**Simpson**	**Fisher**	**Invsimpson**
	**FDR *p***	**FDR *p***	**FDR *p***	**FDR *p***	**FDR *p***	**FDR *p***	**FDR *p***
Lozupone et al. (2013)	0.041^*^	0.049^*^	0.041^*^	0.750	0.750	0.580	0.640
Dillon et al. (2014)	0.078	0.078	0.078	0.078	0.078	0.078	0.078
Dinh et al. (2015)	0.970	0.970	0.970	0.770	0.970	0.770	0.800
Vázquez-Castellanos et al. (2015)	0.020^*^	0.027^*^	0.020^*^	0.027^*^	0.160	0.020^*^	0.014^*^
Dubourg et al. (2016)	0.000^***^	0.000^***^	0.000^***^	0.000^***^	0.003^**^	0.320	0.000^***^
Noguera-Julian et al. (2016)	0.003^**^	0.003^**^	0.003^**^	0.021^*^	0.092	0.003^**^	0.272
Vesterbacka et al. (2017)	0.000^***^	0.000^***^	0.000^***^	0.001^**^	0.012^*^	0.007^**^	0.028^*^
Armstrong et al. (2018)	0.990	0.990	0.990	0.990	0.550	0.000^***^	0.680
Cook et al. (2019)	0.850	0.850	0.850	0.850	0.850	0.850	0.850
Lee et al. (2018)	0.550	0.550	0.550	0.690	0.550	0.550	0.550
Neff et al. (2018)	0.970	0.970	0.970	0.770	0.770	0.770	0.770
Li et al. (2019)	0.990	0.990	0.990	0.990	0.990	0.990	0.990

* *FDR p < 0.05*,

** FDR p < 0.01, and

*** *FDR p < 0.001*.

#### Restricting the Analysis to Gender, Sexual Orientation, Age, and BMI

The subgroup analysis controlled for gender, including 472 men and 175 women. In men, there was no significant difference in alpha diversity between HIV+ and HIV– individuals (Supplementary Figure 2). Among women, HIV+ status was associated with a significant decrease in alpha diversity, including Observed (*Z* = 2.78, FDR *p* = 0.035) and ACE (*Z* = 2.52, FDR *p* = 0.035) (Figure 2b, Supplementary Figure 3). In MSM (*n* = 316), there was no significant difference in alpha diversity between HIV+ and HIV– individuals (Supplementary Figure 4). In non-MSM (*n* = 288), HIV+ status was associated with a significant decrease in alpha diversity, including Observed (*Z* = 3.32, FDR *p* = 0.006), Chao1 (*Z* = 2.69, FDR *p* = 0.010), ACE (*Z* = 2.89, FDR *p* = 0.010), Shannon (*Z* = 2.89, FDR *p* = 0.010), and Simpson (*Z* = 2.81, FDR *p* = 0.009) (Figure 2c, Supplementary Figure 5). When age (age <45 years, *n* = 409; age ≥45 years, *n* = 288) and BMI (BMI = 18.5–23.9, *n* = 206; BMI = 24–27.9, *n* = 201; BMI > 28, *n* = 133) (Supplementary Figures 6–10) were controlled, there was no significant difference in alpha diversity between HIV+ and HIV– individuals. The results after the sensitivity analysis remained consistent.

#### Restricting the Analysis to CD4^+^T Cell Count, ART, and HIV Viral Load

The subgroup analysis controlled for the CD4^+^T cell count, including 146 HIV+ individuals with a CD4^+^T cell count of <500, 272 HIV+ individuals with a CD4^+^T cell count of ≥500, and 217 HIV– individuals. HIV+ individuals with a CD4^+^T cell count of <500 have significantly lower alpha diversity than HIV– individuals (Shannon [*Z* = 2.78, FDR *p* = 0.035]). However, there is no significant difference in the alpha diversity of HIV+ individuals with a CD4^+^T cell count of ≥500 compared to HIV– individuals (Figure 2d, Supplementary Figures 11, 12). When controlled for the ART, including 188 HIV+ non-ART users, 240 HIV+ ART users, and 294 HIV– individuals. The results showed that the alpha diversity of HIV+ non-ART users was significantly lower than that of HIV– individuals (Shannon [*Z* = 2.91, FDR *p* = 0.021]), and the difference was not found in HIV+ ART users and HIV– individuals (Figure 2e, Supplementary Figures 13, 14). In the stratified analyses examining the HIV VL (VL ≤ 200, *n* = 244; VL > 200, *n* = 174; and HIV– individuals, *n* = 217) was not significantly associated with alpha diversity (Supplementary Figures 15, 16). The results after the sensitivity analysis remained consistent.

### Richness and Diversity of the Gut Microbiome Based on MSM Status

**Figure 4:**
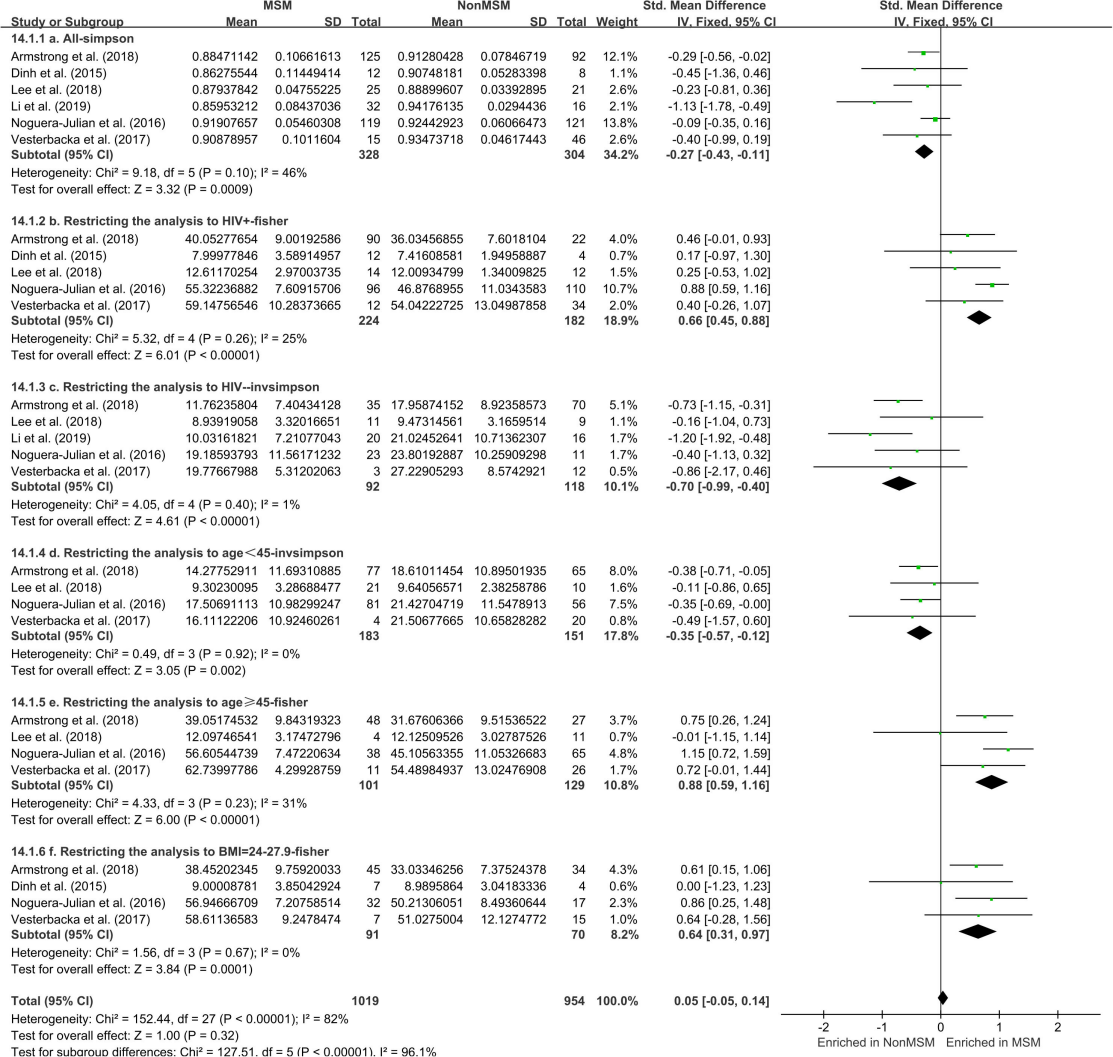
Forest plots comparing MSM to non-MSM (NonMSM) individuals. Before controlling for other confounding factors, the alpha diversity of the MSM was significantly lower than that of the non-MSM individuals (**a**, Simpson index). When restricting the analysis to HIV+ (**b**, Fisher index), age ≥45 years (**e**, Fisher index), BMI = 24–27.9 (**f**, Fisher index) individuals, MSM status was associated with an increase in alpha diversity. When restricting the analysis to HIV– individuals (**c**, Invsimpson index) and age <45 years (d, Invsimpson index), MSM status was associated with a significant decrease in alpha diversity.

**Figure 5:**
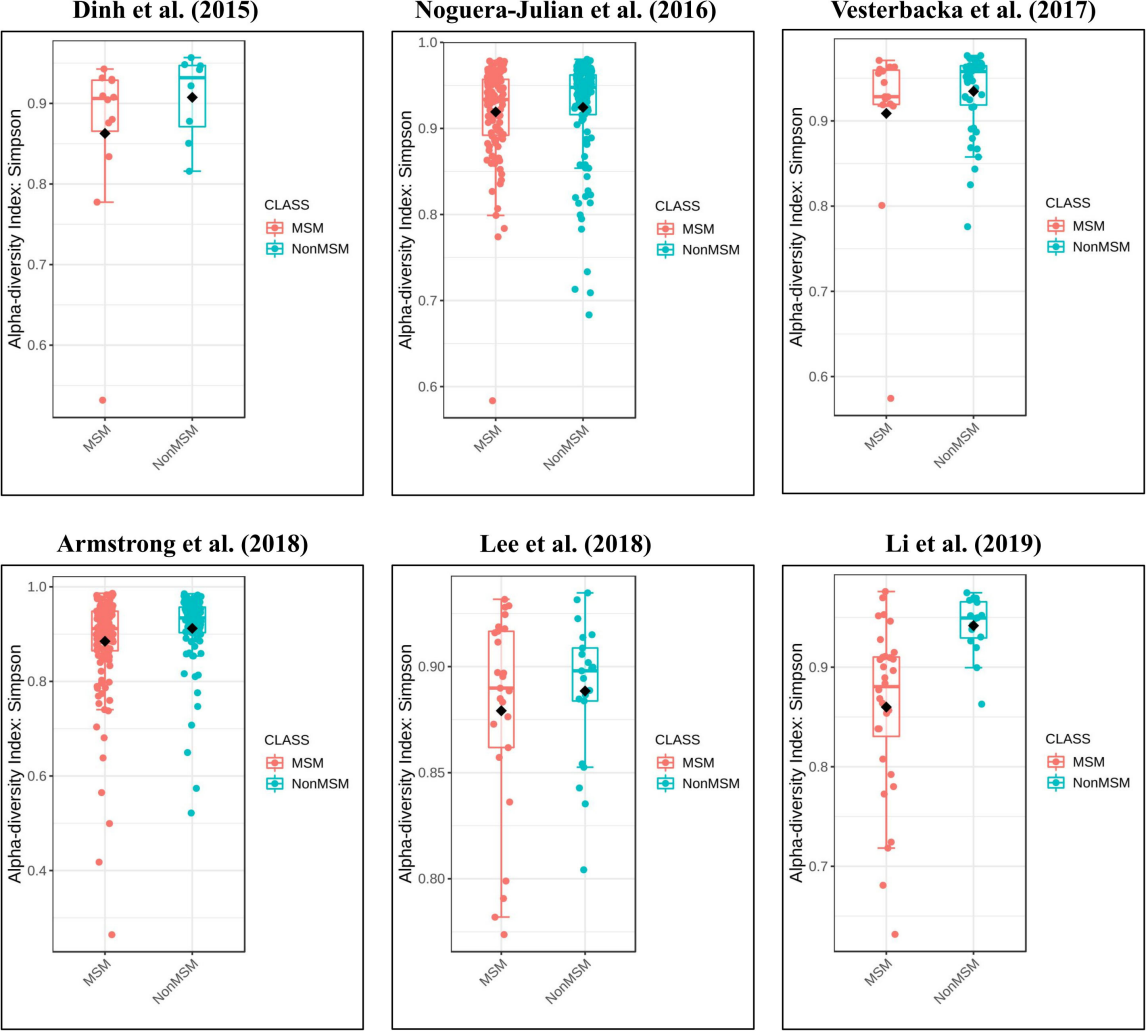
Boxplots showing the alpha diversity in terms of the Simpson index by study and MSM status (red, MSM; blue, non-MSM). All studies showed decreased alpha diversity in MSM compared to non-MSM.

The calculation results of the alpha diversity indexes of the OTU level of six studies are shown in Table 3. Before controlling for other confounding factors, the alpha diversity of the MSM individuals was significantly lower than that of the non-MSM individuals, including the Simpson (*Z* = 3.32, FDR *p* = 0.001) and Invsimpson (*Z* = 3.49, FDR *p* = 0.001) of the FEM. The forest maps (Figure 4a, Supplementary Figure 17) and boxplots (Figure 5) of the six studies included also show this trend. The analysis was restricted to HIV status (HIV+ individuals, *n* = 406; HIV– individuals, *n* = 210), age (age <45 years, *n* = 334; age ≥45 years, *n* = 230), and BMI (BMI = 18.5–23.9, *n* = 164; BMI = 24–27.9, *n* = 161; BMI > 28, *n* = 96). In the HIV+ (Fisher [*Z* = 6.01, FDR *p* < 0.000]), age ≥45 years (Fisher [*Z* = 6, FDR *p* < 0.000]), and BMI = 24–27.9 (Fisher [*Z* = 3.84, FDR *p* = 0.001]) (Figures 4b,e,f, Supplementary Figures 18, 21–24) individuals, MSM status was associated with increasing alpha diversity. Among HIV– (Invsimpson [*Z* = 4.61, FDR *p* < 0.000]) and age <45 years individuals (Invsimpson [*Z* = 3.05, FDR *p* = 0.005]), MSM status was associated with a significant decrease in alpha diversity (Figures 4c,d, Supplementary Figures 19, 20). The results after the sensitivity analysis remained consistent.

**Table 3:** The alpha diversity results of MSM in OTU level.

**References**	**Observed**	**Chao1**	**ACE**	**Shannon**	**Simpson**	**Fisher**	**Invsimpson**
	**FDR *p***	**FDR *p***	**FDR *p***	**FDR *p***	**FDR *p***	**FDR *p***	**FDR *p***
Dinh et al. (2015)	0.660	0.750	0.660	0.630	0.630	0.660	0.630
Noguera-Julian et al. (2016)	0.000^***^	0.000^***^	0.000^***^	0.190	0.480	0.000^***^	0.110
Vesterbacka et al. (2017)	0.630	0.630	0.630	0.630	0.630	0.630	0.630
Armstrong et al. (2018)	0.880	0.700	0.700	0.190	0.098	0.015^*^	0.300
Lee et al. (2018)	0.590	0.590	0.590	0.930	0.630	0.590	0.840
Li et al. (2019)	0.340	0.840	0.840	0.000^***^	0.000^***^	0.460	0.000^***^

* *FDR p < 0.05*,

** FDR p < 0.01, and

*** *FDR p < 0.001*.

### Composition of the Gut Microbiome Associated With HIV+ and MSM Status

**Figure 6:**
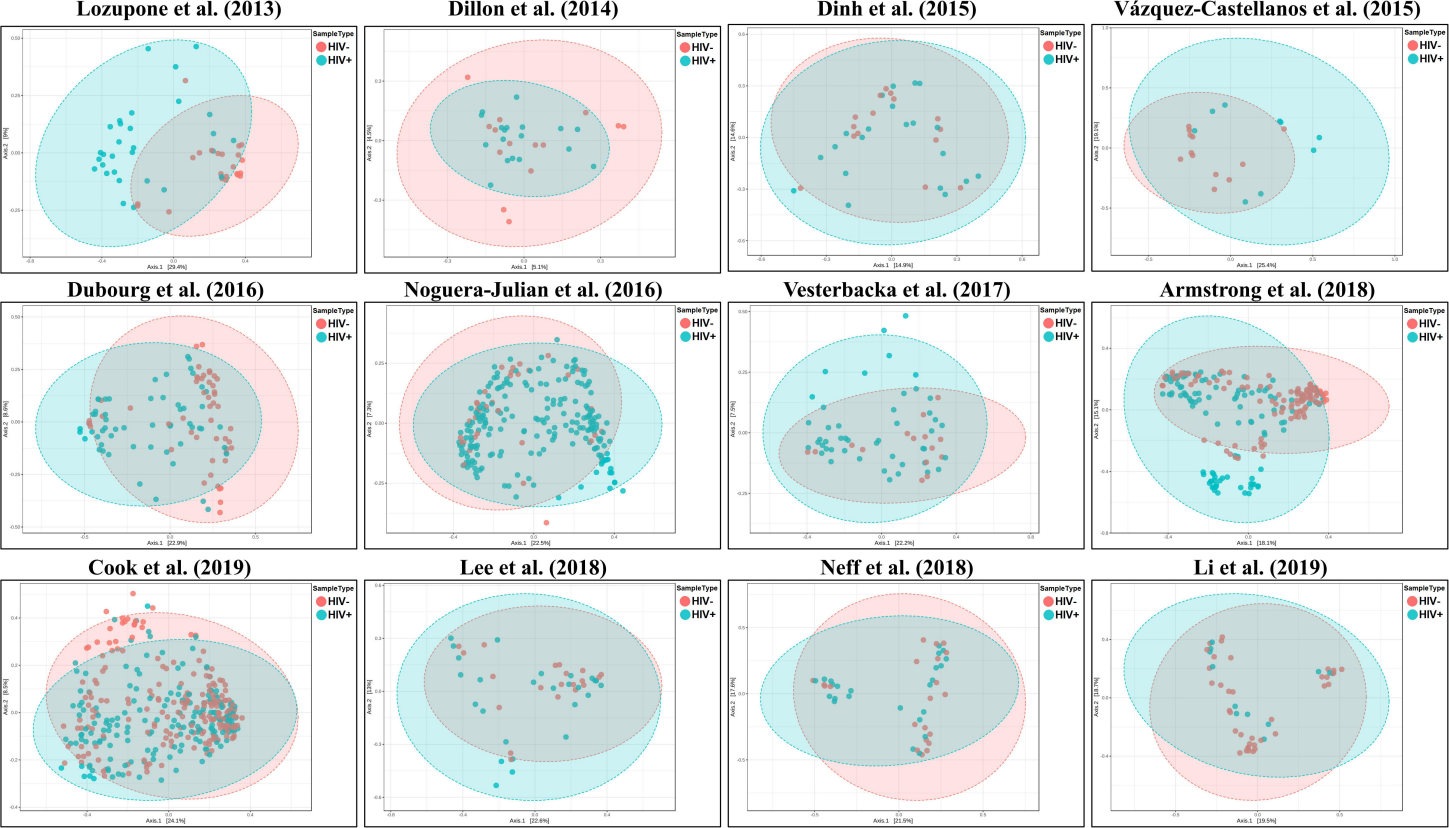
The PCoA ordination plots by study and HIV status. PCoA ordination plots of Bray–Curtis showing that the microbiomes in the datasets by Lozupone et al. (2013) (*R*^2^ = 0.154, FDR *p* < 0.001), Dubourg et al. (2016) (*R*^2^ = 0.080, FDR *p* < 0.001), and Armstrong et al. (2018) (*R*^2^ = 0.059, FDR *p* < 0.001) showed better cluster according HIV status.

**Figure 7:**
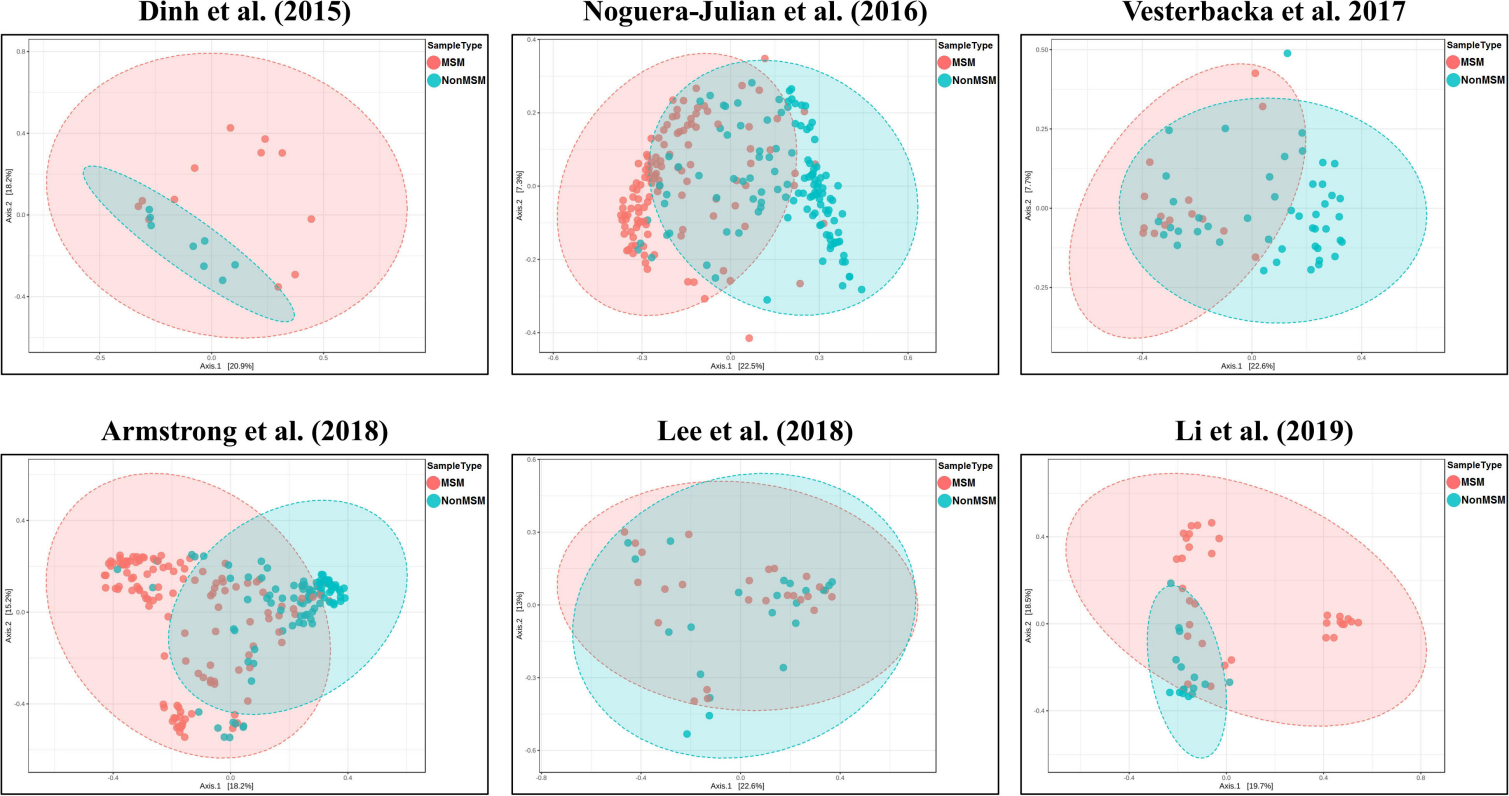
The PCoA ordination plots by study and MSM status. PCoA ordination plots of Bray–Curtis of Noguera-Julian et al. (2016) (*R*^2^ = 0.122, FDR *p* < 0.001), Vesterbacka et al. (2017) (*R*^2^ = 0.081, FDR *p* < 0.001), Armstrong et al. (2018) (*R*^2^ = 0.090, FDR *p* < 0.001), and Li et al. (2019) (*R*^2^ = 0.115, FDR *p* < 0.001) showed better cluster according to MSM status.

We explored the potential influence of HIV and MSM status on the composition of the gut microbiome, according to the PERMANOVA test of ecological distances. PCoA ordination plots of Bray–Curtis showed that samples from works by Lozupone et al. (2013) (*R*^2^ = 0.154, FDR *p* < 0.001), Dubourg et al. (2016) (*R*^2^ = 0.080, FDR *p* < 0.001), and Armstrong et al. (2018) (*R*^2^ = 0.059, FDR *p* < 0.001) were significantly clustered according to HIV status (Figure 6, Table 4). The samples from works by Noguera-Julian et al. (2016) (*R*^2^ = 0.122, FDR *p* < 0.001), Vesterbacka et al. (2017) (*R*^2^ = 0.081, FDR *p* < 0.001), Armstrong et al. (2018) (*R*^2^ = 0.090, FDR *p* < 0.001), and Li et al. (2019) (*R*^2^ = 0.115, FDR *p* < 0.001) showed better clustering according to MSM status rather than HIV status (Figure 7, Table 5).

**Table 4:** The beta diversity results of HIV in OTU level.

**References**	**Bray-Curtis Index**			**Jensen-Shannon Divergence**			**Jaccard Index**		
	***F***	***R*^**2**^**	**FDR *p***	***F***	***R*^**2**^**	**FDR *p***	***F***	***R*^**2**^**	**FDR *p***
Lozupone et al. (2013)	9.131	0.154	<0.001^***^	14.016	0.219	<0.001^***^	6.167	0.110	<0.001^***^
Dillon et al. (2014)	0.515	0.017	<1.000	0.301	0.010	<1.000	0.748	0.024	<1.000
Dinh et al. (2015)	0.992	0.028	<0.460	1.012	0.029	<0.460	1.004	0.029	<0.460
Vázquez-Castellanos et al. (2015)	2.691	0.124	<0.012^*^	3.631	0.160	<0.012^*^	2.096	0.099	<0.012^*^
Dubourg et al. (2016)	9.073	0.080	<0.001^***^	13.751	0.117	<0.001^***^	5.916	0.054	<0.001^***^
Noguera-Julian et al. (2016)	2.821	0.012	<0.002^**^	4.123	0.017	<0.002^**^	2.150	0.009	<0.002^**^
Vesterbacka et al. (2017)	2.052	0.033	<0.021^*^	2.477	0.040	<0.021^*^	1.682	0.027	<0.021^*^
Armstrong et al. (2018)	13.380	0.059	<0.001^***^	20.417	0.087	<0.001^***^	8.695	0.039	<0.001^***^
Cook et al. (2019)	3.489	0.009	<0.001^***^	5.237	0.014	<0.001^***^	2.421	0.006	<0.001^***^
Lee et al. (2018)	1.002	0.022	<0.430	1.059	0.024	<0.430	1.013	0.022	<0.430
Neff et al. (2018)	1.032	0.023	<0.390	1.079	0.024	<0.390	1.004	0.023	<0.390
Li et al. (2019)	0.987	0.021	<0.430	0.962	0.020	<0.430	1.014	0.022	<0.430

* *FDR p < 0.05*,

** FDR p < 0.01, and

*** *FDR p < 0.001*.

**Table 5:** The beta diversity results of MSM in OTU level.

**References**	**Bray-Curtis Index**			**Jensen-Shannon Divergence**			**Jaccard Index**		
	***F***	***R*^**2**^**	**FDR *p***	***F***	***R*^**2**^**	**FDR *p***	***F***	***R*^**2**^**	**FDR *p***
Dinh et al. (2015)	1.838	0.093	<0.020^*^	2.362	0.116	<0.020^*^	1.531	0.078	<0.020^*^
Noguera-Julian et al. (2016)	32.974	0.122	<0.001^***^	55.313	0.189	<0.001^***^	19.926	0.077	<0.001^***^
Vesterbacka et al. (2017)	5.225	0.081	<0.001^***^	8.669	0.128	<0.001^***^	3.573	0.057	<0.001^***^
Armstrong et al. (2018)	21.148	0.090	<0.001^***^	32.809	0.132	<0.001^***^	13.312	0.058	<0.001^***^
Lee et al. (2018)	1.523	0.033	<0.099	1.712	0.037	<0.099	1.385	0.031	<0.099
Li et al. (2019)	5.990	0.115	<0.001^***^	8.281	0.153	<0.001^***^	4.059	0.081	<0.001^***^

* *FDR p < 0.05*,

** *FDR p < 0.01*,

*** *FDR p < 0.001*.

**Figure 8:**
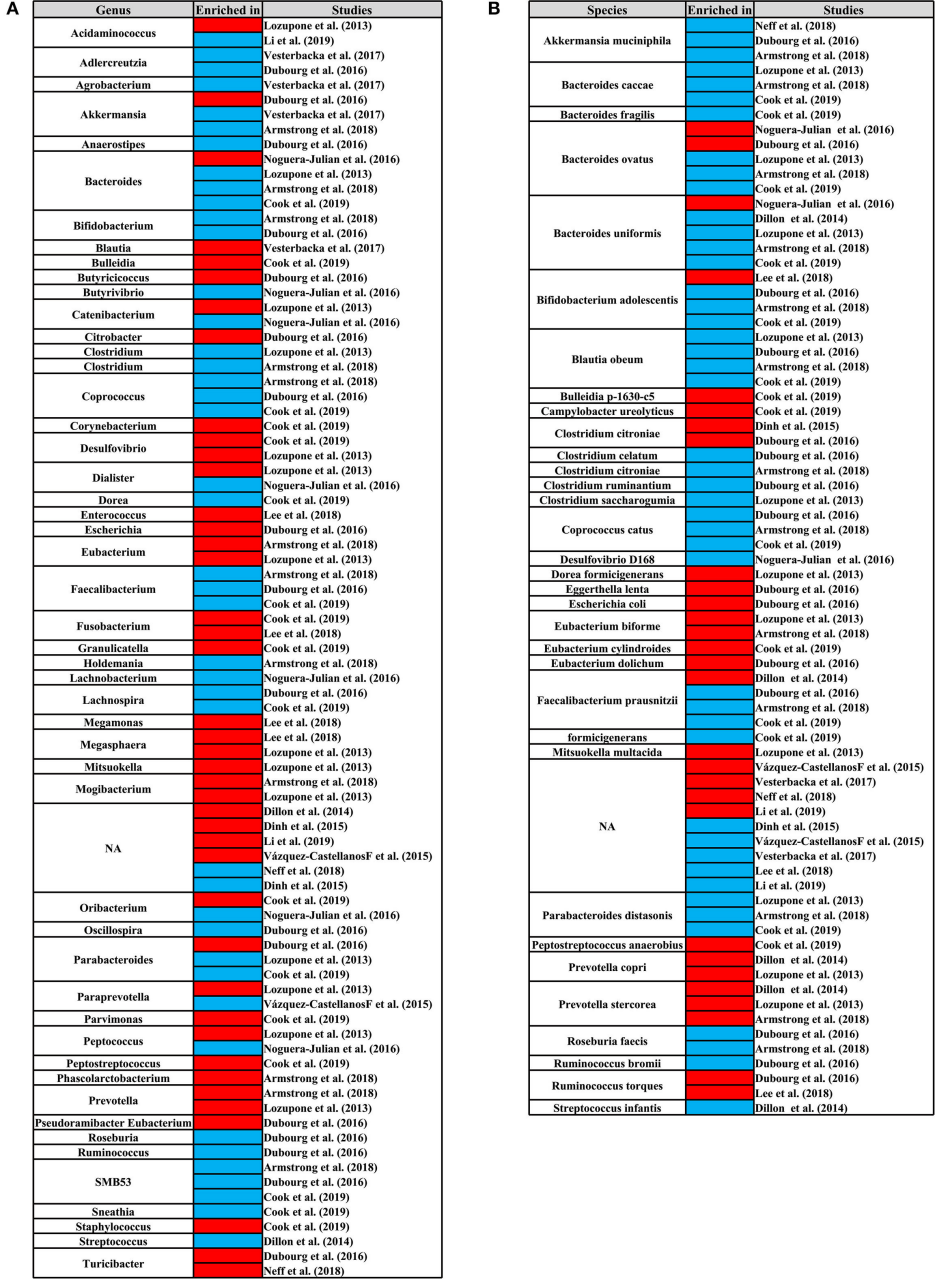
Differential genus **(A)** and species **(B)** map related to HIV status. From left to right, the first column represents the differential genus or species, and the third column represents the studies. The third column represents the research corresponding to the differential genus or species. The second column shows the differential enrichment of genus or species in HIV+ and HIV– individuals. Red indicates that the genus or species were significantly enriched in HIV+ individuals. Blue indicates that the genus or species were significantly enriched in HIV– individuals.

**Figure 9:**
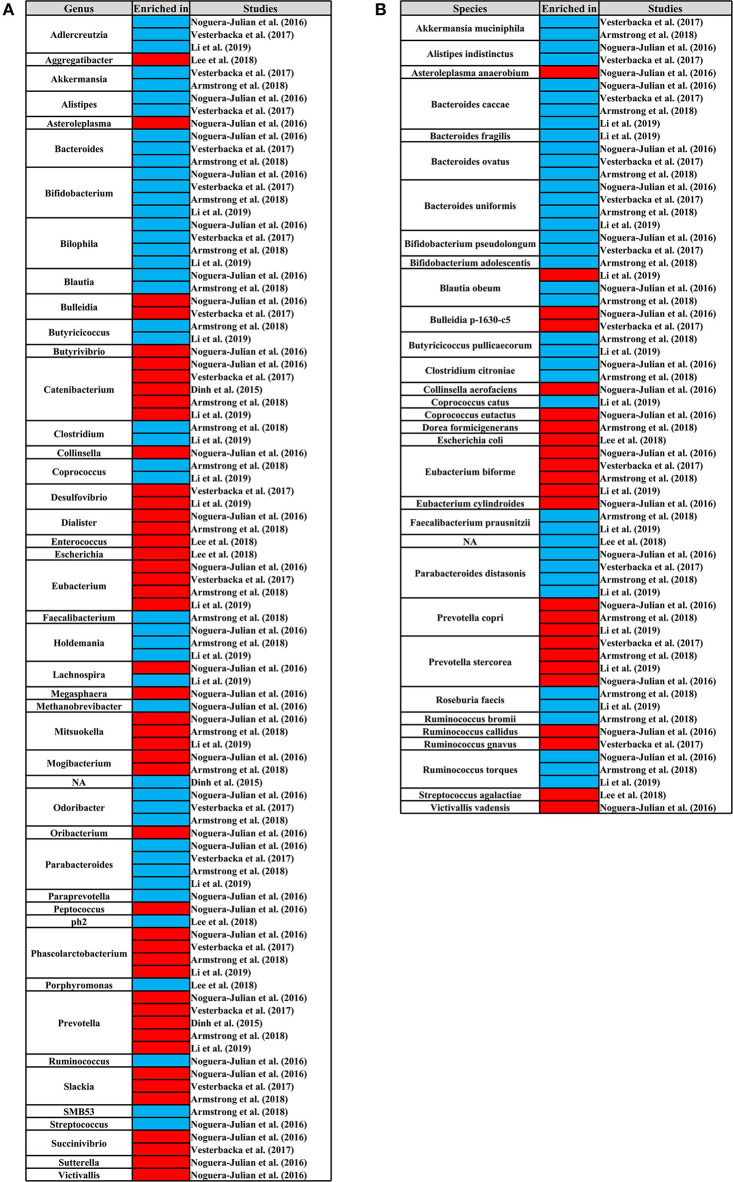
Differential genus **(A)** and species **(B)** map related to MSM status. From left to right, the first column represents the differential genus or species, and the third column represents the studies. The third column represents the research corresponding to the differential genus or species. The second column shows the differential enrichment of genus or species in MSM and non-MSM. Red indicates that the genus or species were significantly enriched in MSM. Blue indicates that the genus or species were significantly enriched in non-MSM.

We identified the genus and species that cause changes in the composition of the gut microbiome of HIV+ individuals and MSM. In at least three studies, the genus of *Bacteroides, Coprococcus, Faecalibacterium*, and *SMB53* and the species of *Bifidobacterium adolescentis, Bacteroides caccae, Coprococcus catus, Parabacteroides distasonis, Akkermansia muciniphila, Blautia obeum, Bacteroides ovatus, Faecalibacterium prausnitzii*, and *Bacteroides uniformis* were significantly reduced in HIV+ individuals. The species of *Prevotella stercorea* was significantly increased in HIV+ individuals (Figure 8). In MSM, the genus of *Catenibacterium, Eubacterium, Mitsuokella, Phascolarctobacterium, Prevotella*, and *Slackia* and the species of *Eubacterium biforme, Prevotella copri*, and *Prevotella stercorea* were significantly increased, and the genus of *Adlercreutzia, Bacteroides, Bifidobacterium, Bilophila, Holdemania, Odoribacter, Parabacteroides* and the species of *Bacteroides caccae, Parabacteroides distasonis, Bacteroides ovatus, Ruminococcus torques*, and *Bacteroides uniformis* were significantly reduced (Figure 9).

### Differential KEGG Functional Pathway Analysis

**Figure 10:**
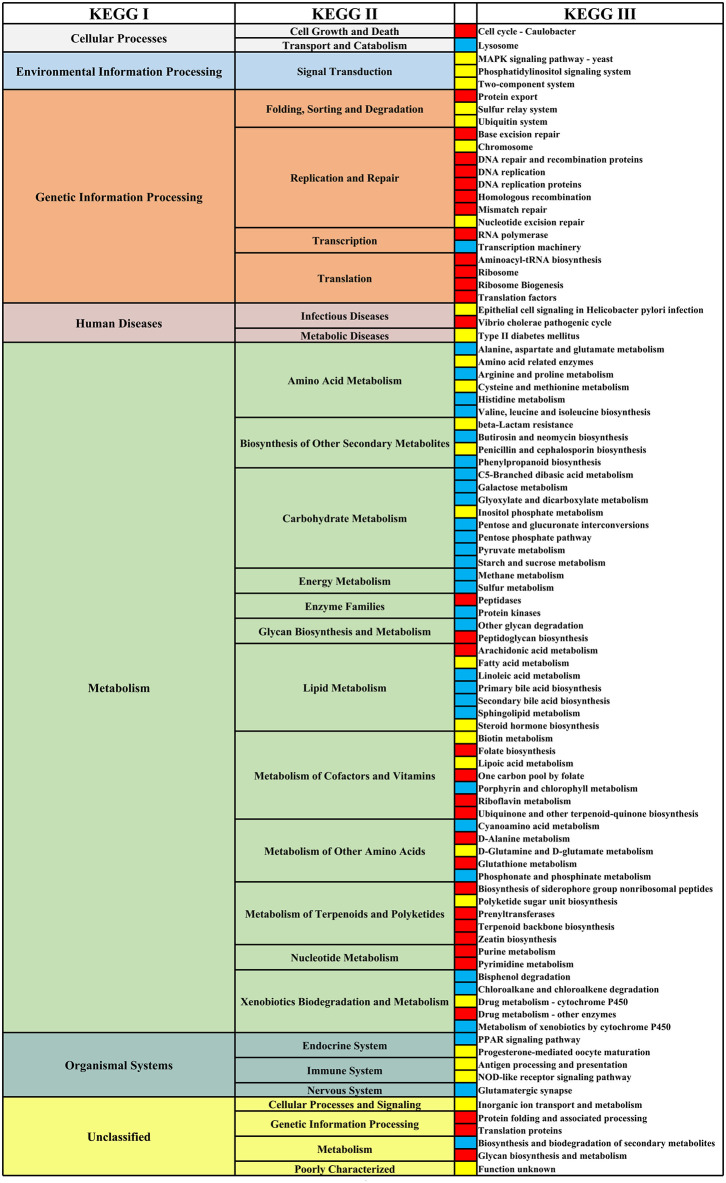
Differential KEGG functional pathways map related to HIV status. From left to right, the first column represents the KEGG I functional pathways, and the second and fourth columns represent the KEGG II and KEGG III functional pathways, respectively, under KEGG I. The third column shows the differential enrichment of the KEGG III functional pathways in HIV+ and HIV– individuals. Red indicates that the functional pathways were significantly enriched in HIV+ individuals in at least three studies. Blue indicates that the functional pathways were significantly enriched in HIV– individuals in at least three studies. Yellow indicates that the abundances of the functional pathways were significantly different in at least three studies, but the direction of changes related to HIV status was inconsistent.

**Figure 11:**
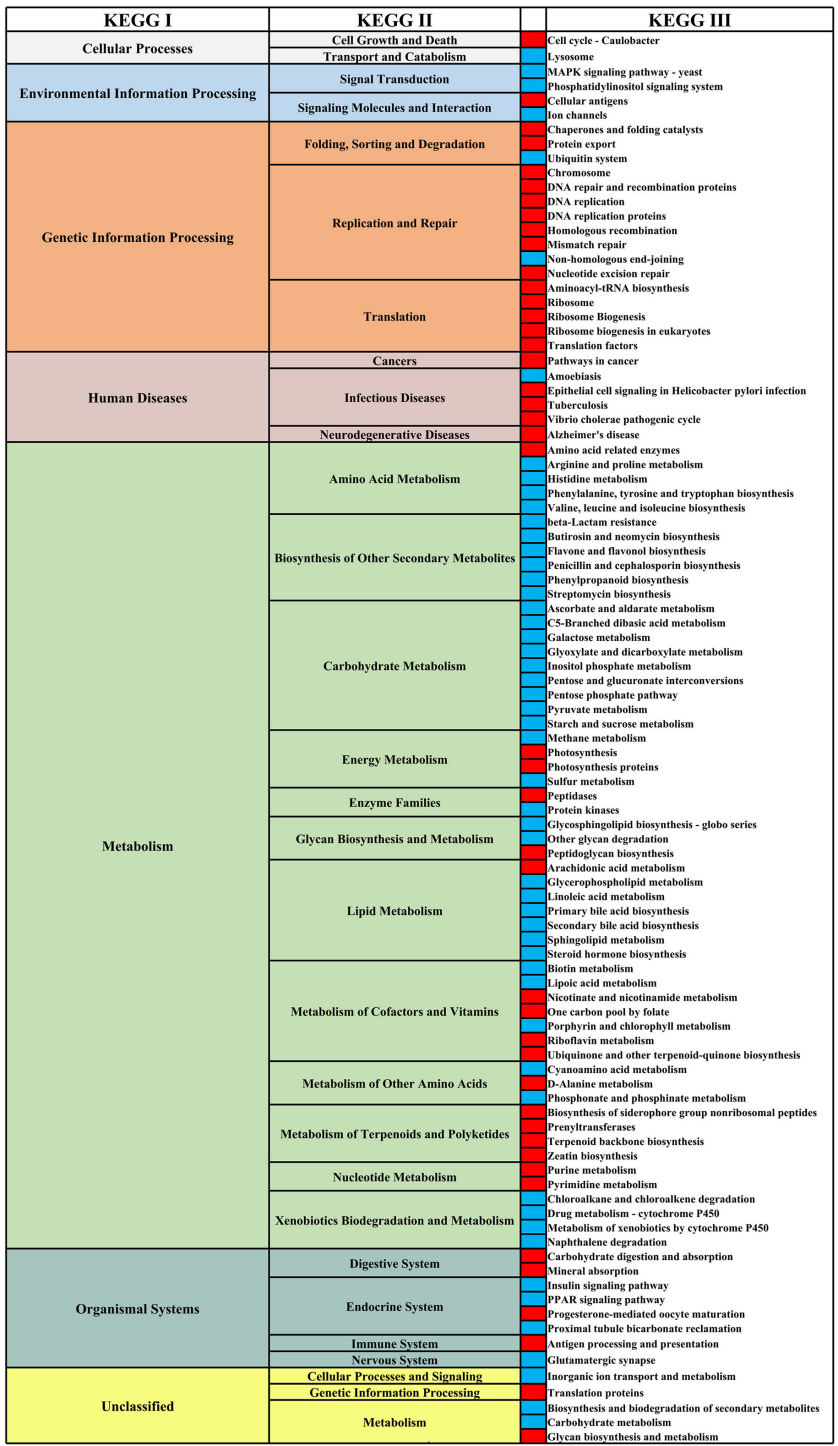
Differential KEGG functional pathways map related to MSM status. From left to right, the first column represents the KEGG I functional pathways, and the second and fourth columns represent the KEGG II and KEGG III functional pathways, respectively, under KEGG I. The third column shows the differential enrichment of the KEGG III functional pathways in MSM and non-MSM individuals. Red indicates that the functional pathways were significantly enriched in MSM in at least three studies. Blue indicates that the functional pathways were significantly enriched in non-MSM in at least three studies.

We analyzed the differential functional pathways of KEGG related to HIV and MSM status. For HIV status, there were 91 differential KEGG III functional pathways that co-exist in multiple studies (≥3 studies). For MSM status, there were 97 differential KEGG functional pathways that co-exist in multiple studies (≥3 studies). Among HIV+ individuals and MSM, most of the KEGG III pathways under metabolism were downregulated, and most of the KEGG III pathways under genetic information processing were downregulated (Figures 10, 11). For example, in HIV+ individuals and MSM, carbohydrate-metabolism-related pathways of galactose metabolism, pyruvate metabolism, and the pentose phosphate pathway, were downregulated; amino-acid-metabolism-related pathways of histidine metabolism, arginine and proline metabolism, and valine, leucine, and isoleucine biosynthesis, were downregulated; and lipid-metabolism-related pathways of linoleic acid metabolism, primary bile acid biosynthesis, and secondary bile acid biosynthesis, were downregulated. The prediction accuracy of different PICRUSt studies related to HIV and MSM status are shown in Supplementary Table 2 and Table 3.

## Discussion

In this study, we collected 12 studies to evaluate the relationship between the gut microbiome and the HIV and MSM status. In the overall assessment of 12 datasets and by restricting the analysis to woman and non-MSM individuals, HIV+ status was associated with decreased alpha diversity, consistent with the results of a recent meta-analysis (Tuddenham et al., 2020). Importantly, when controlling for a CD4^+^T cell count of <500 and non-ART, HIV+ status was also significantly associated with decreased alpha diversity. The assessment of the overall effect of six datasets related to MSM status showed that MSM status was associated with decreased alpha diversity, but the results of the subgroup analysis (restricting the analysis to HIV status, age <45 years, age ≥45 years, BMI = 24–27.9) were inconsistent. The analysis of the microbiome composition showed that in multiple studies the sample clustered in different areas of the PCoA coordinate axis according to HIV and MSM status. This clustering phenomenon is more significant between MSM and non-MSM. We also found that HIV+ and MSM status were related to consistent changes in the specific genera, species, and KEGG functional pathways.

In recent years, consistent alpha diversity of the gut microbiome associated with HIV infection has not been clarified (Lozupone et al., 2013; Mutlu et al., 2014; Dinh et al., 2015; Dubourg et al., 2016; Nowak et al., 2017). Therefore, we assembled the largest dataset to date to evaluate the alpha diversity of the gut microbiome related to HIV status. Whether in the overall effect analysis based on 12 studies or in the subgroup analysis, our results indicated that HIV+ status was associated with decreased alpha diversity of the gut microbiome, which was consistent with most current research results (Mutlu et al., 2014; Nowak et al., 2015; Dubourg et al., 2016; Noguera-Julian et al., 2016; Vesterbacka et al., 2017; Tuddenham et al., 2020). In our subgroup analysis, the downregulation of alpha diversity in the gut microbiome is related to HIV+ non-ART status, consistent with the results by Vesterbacka et al. (2017). However, there was evidence that ART also induces changes in the gut microbiome, unrelated to HIV infection. Some authors have implied that ART may enhance dysbiosis, which is consistent with the high frequency of gastrointestinal symptoms associated with ART (Lozupone et al., 2014; Nowak et al., 2015; Noguera-Julian et al., 2016). In addition, severe mucosal CD4^+^T cell depletion is an important reason for disruption of the gut epithelial barrier and translocation of the gut microbiome in the early stage of HIV infection (Hirao et al., 2014). We also confirmed that the CD4^+^T cell depletion in HIV+ individuals is closely related to the gut microbiome.

Another group of people that we evaluated was MSM. Recent studies have shown that MSM status may profoundly affect the structure of the gut microbiome, which may be stronger than HIV, and this factor may confound many studies of HIV-related gut microbiomes (Noguera-Julian et al., 2016; Kelley et al., 2017; Armstrong et al., 2018; Neff et al., 2018; Guillen et al., 2019; Hensley-McBain et al., 2019; Kehrmann et al., 2019; Li et al., 2019). In our study, a significant reduction in alpha diversity associated with HIV+ status was found in non-MSM individuals, not MSM. Further analysis revealed that there was a significant difference in the gut microbiome alpha diversity between MSM and non-MSM individuals and samples were better clustered in PCoA by MSM, rather than HIV status. These trends reflect previously published results showing that the gut microbiome of MSM has higher immune activity than men who have sex with women (MSW), regardless of HIV infection (Neff et al., 2018; Li et al., 2019).

In addition, many cross-sectional studies have indicated that the gut microbiome shifts from *Bacteroides* to *Prevotella* predominance after HIV infection (Lozupone et al., 2013; Mutlu et al., 2014; Vázquez-Castellanos et al., 2015; Ling et al., 2016; Dillon et al., 2017; Serrano-Villar et al., 2017). However, the latest research suggests that the *Prevotella* predominance is associated with MSM rather than HIV status (Armstrong et al., 2018; Neff et al., 2018; Li et al., 2019). For the inconsistent results of the previous studies, we used LEfSe method to identify the differential genus related to HIV+ and MSM status and used DESeq2 and Random Forests method to verify. Our results showed that in multiple studies (≥3 studies), HIV+ status was related to the abundant downregulation of *Bacteroides, Coprococcus, Faecalibacterium*, and *SMB53*, while the *Prevotella*-rich and *Bacteroides*-poor were more closely related to MSM status. For the *Prevotella*-rich not seen in HIV+ individuals, a study found that *Prevotella* abundance decreased after ART initiation (Nowak et al., 2015). It is worth noting that at the species level, the pattern of microbial composition with decreased abundance of *B. caccae, B. ovatus*, and *B. uniformis* and increased abundance of *P. stercorea* is consistent in HIV+ individuals and MSM. *Prevotella* spp. is generally considered to have pro-inflammatory effects, whereas *Bacteroides* spp. has a role in promoting T-regulatory cell function. For example, studies have found evidence that enhanced CD4^+^T cell HIV infection or inflammation induction is associated with experiments utilizing *P. copri* or *P. stercorea* (Dillon et al., 2016; Kaur et al., 2018). *Bacteroides* is considered to be the main genus of the core microbiome module; of which, the species with relative abundances exceeding 1% are *B. uniformis, B. vulgatus, B. caccae*, and *B. thetaiotaomicron* (Tan et al., 2019). Gauffin Cano et al. demonstrated that the *B. uniformis CECT7771* is capable of ameliorating the overweight-associated immune dysfunctions (Gauffin Cano et al., 2012). Hamady et al. (2010, 2011) found that the *B. ovatus* can prevent colitis caused by DSS in the form of improving weight loss and reducing the colon length, and downregulating the secretion of proinflammatory cytokines such as TNF-α, IL-1β, and IL-6 (Hamady et al., 2010, 2011). These findings suggest that MSM status may be an independent factor related to dysbiosis of the gut microbiome. Meanwhile, early regulation of MSM-related gut microbiome dysbiosis is of great significance for the prevention and treatment of HIV infection and intestinal inflammation.

The results of the KEGG functional pathway analysis showed gut microbiome—gut microbiome interactions and gut microbiome—human body interactions. For example, a study using gnotobiotic mouse models showed that extracellular digestion of inulin increases the growth rate of *B. ovatus*. In turn, by-products from inulin catabolism can be used by *F. prausnitzii* and *B. vulgatus* (Rakoff-Nahoum et al., 2016). Our research also confirmed this result. That is, in HIV+ individuals, the abundance of carbohydrate metabolism and *F. prausnitzii* is significantly downregulated with the downregulation of *B. ovatus*. Comparative analysis of microbial genomes shows that more than 98% of all microbiomes (such as *Bacteroides* spp.) sequenced so far lack essential pathways or key genes for amino acid synthesis. Therefore, most microbiomes are auxotrophic and require a source of extracellular amino acids, vitamins, and/or cofactors to survive (Mee and Wang, 2012; Mee et al., 2014). Our research also shows that the reduction in the abundance of *Bacteroides* in HIV+ individuals and MSM was accompanied by the downregulation of amino-acid-metabolism-related pathways.

This study has some limitations. First, we did not collect all demographic and disease characteristics related to the subjects, which leads to a lack of enough datasets for our subgroup analysis. We analyzed the relationship between the gut microbiome and MSM status based on HIV-related data. There are many confounding factors in the analysis related to MSM status, such as age, disease status, and BMI, which are often not matched in HIV-related research, and our included studies are mainly from Europe and the Americas, lacking research from Asia and Africa. Moreover, the shotgun data of the gut microbiome were excluded in our analysis, which might cause flaws in our findings, especially in the bacterial functional pathway analysis. Lastly, the different studies used different variable regions and instruments for 16S rRNA gene amplicon sequencing. Although we have processed and standardized the sequences according to the characteristics of the data, different experimental techniques may still cause bias.

In conclusion, our results clarified that HIV+ status is associated with decreased alpha diversity of the gut microbiome. MSM status was an important factor affecting the study of HIV-related gut microbiomes; that is, MSM was associated with alpha diversity changes in the gut microbiome regardless of HIV infection, and the change in gut microbiome composition of MSM was more significant than that of HIV+ individuals. There was a consistent change in *B. caccae, B. ovatus, B. uniformis*, and *P. stercorea*, in HIV+ individuals and MSM. The differential expression of the gut microbiome was also accompanied by changes in functional pathways, such as carbohydrate metabolism, amino acid metabolism, and lipid metabolism, These findings might help to elucidate the effects of HIV+ and MSM status on the gut microbiome in humans.

## Data Availability Statement

The datasets generated for this study can be found in the bioproject PRJNA227062, PRJEB4335, PRJNA233597, PRJEB5185, PRJEB10578, PRJNA307231, PRJNA354863, PRJEB28485, PRJNA422134, PRJNA489590, PRJEB25418, and PRJEB31328.

## Author Contributions

JH, HL, and LY designed the study. JZ, YZ, PC, LL, HC, BL, JJ, CN, LT, and XZ participated in data acquisition. JZ contributed to data analysis. JZ, JH, and YZ participated in interpreting the results and preparing the report for publication. All authors revised the manuscripts critically and approved the final version for publication.

## Conflict of Interest

The authors declare that the research was conducted in the absence of any commercial or financial relationships that could be construed as a potential conflict of interest.

## References

[B1] Armstrong A. J. S. Shaffer M. Nusbacher N. M. Griesmer C. Fiorillo S. Schneider J. M.. (2018). An exploration of *Prevotella*-rich microbiomes in HIV and men who have sex with men. Microbiome 6:198. 10.1186/s40168-018-0580-7 30396369 PMC6219090

[B2] Balagopal A. Philp F. H. Astemborski J. Block T. M. Mehta A. Long R.. (2008). Human immunodeficiency virus-related microbial translocation and progression of hepatitis C. Gastroenterology 135, 226–233. 10.1053/j.gastro.2008.03.022 18457674 PMC2644903

[B3] Brenchley J. M. Price D. A. Schacker T. W. Asher T. E. Silvestri G. Rao S.. (2006). Microbial translocation is a cause of systemic immune activation in chronic HIV infection. Nat. Med. 12, 1365–1371. 10.1038/nm1511 17115046

[B4] Caporaso J. G. Kuczynski J. Stombaugh J. Bittinger K. Bushman F. D. Costello E. K.. (2010). QIIME allows analysis of high-throughput community sequencing data. Nat. Methods 7, 335–336. 10.1038/nmeth.f.303 20383131 PMC3156573

[B5] Cook R. R. Fulcher J. A. Tobin N. H. Li F. Lee D. Javanbakht M.. (2019). Effects of HIV viremia on the gastrointestinal microbiome of young MSM. AIDS 33, 793–804. 10.1097/QAD.0000000000002132 30649052 PMC6422698

[B6] DeSantis T. Z. Hugenholtz P. Larsen N. Rojas M. Brodie E. L. Keller K.. (2006). Greengenes, a chimera-checked 16S rRNA gene database and workbench compatible with ARB. Appl. Environ. Microbiol. 72, 5069–5072. 10.1128/AEM.03006-05 16820507 PMC1489311

[B7] Dhariwal A. Chong J. Habib S. King I. L. Agellon L. B. Xia J. (2017). MicrobiomeAnalyst: a web-based tool for comprehensive statistical, visual and meta-analysis of microbiome data. Nucleic Acids Res. 45, 180–188. 10.1093/nar/gkx295 28449106 PMC5570177

[B8] Dillon S. M. Kibbie J. Lee E. J. Guo K. Santiago M. L. Austin G. L.. (2017). Low abundance of colonic butyrate-producing bacteria in HIV infection is associated with microbial translocation and immune activation. AIDS 31, 511–521. 10.1097/QAD.0000000000001366 28002063 PMC5263163

[B9] Dillon S. M. Lee E. J. Donovan A. M. Guo K. Harper M. S. Frank D. N.. (2016). Enhancement of HIV-1 infection and intestinal CD4+ T cell depletion *ex vivo* by gut microbes altered during chronic HIV-1 infection. Retrovirology 13:5. 10.1186/s12977-016-0237-1 26762145 PMC4712466

[B10] Dillon S. M. Lee E. J. Kotter C. V. Austin G. L. Dong Z. Hecht D. K.. (2014). An altered intestinal mucosal microbiome in HIV-1 infection is associated with mucosal and systemic immune activation and endotoxemia. Mucosal Immunol. 7, 983–994. 10.1038/mi.2013.116 24399150 PMC4062575

[B11] Dinh D. M. Volpe G. E. Duffalo C. Bhalchandra S. Tai A. K. Kane A. V.. (2015). Intestinal microbiota, microbial translocation, and systemic inflammation in chronic HIV infection. J. Infect. Dis. 211, 19–27. 10.1093/infdis/jiu409 25057045 PMC4326316

[B12] Dubourg G. Lagier J. C. Hue S. Surenaud M. Bachar D. Robert C.. (2016). Gut microbiota associated with HIV infection is significantly enriched in bacteria tolerant to oxygen. BMJ Open Gastroenterol. 3:e000080. 10.1136/bmjgast-2016-000080 27547442 PMC4985784

[B13] Edgar R. C. (2010). Search and clustering orders of magnitude faster than BLAST. Bioinformatics 26, 2460–2461. 10.1093/bioinformatics/btq461 20709691

[B14] Edgar R. C. Haas B. J. Clemente J. C. Quince C. Knight R. (2011). UCHIME improves sensitivity and speed of chimera detection. Bioinformatics 27, 2194–2200. 10.1093/bioinformatics/btr381 21700674 PMC3150044

[B15] Epple H. J. Allers K. Troger H. Kuhl A. Erben U. Fromm M.. (2010). Acute HIV infection induces mucosal infiltration with CD4+ and CD8+ T cells, epithelial apoptosis, and a mucosal barrier defect. Gastroenterology 139, 1289–1300. 10.1053/j.gastro.2010.06.065 20600014

[B16] Gauffin Cano P. Santacruz A. Moya A. Sanz Y. (2012). *Bacteroides uniformis* CECT 7771 ameliorates metabolic and immunological dysfunction in mice with high-fat-diet induced obesity. PLoS ONE 7:e41079. 10.1371/journal.pone.0041079 22844426 PMC3406031

[B17] Goecks J. Eberhard C. Too T. Nekrutenko A. Taylor J. (2013). Web-based visual analysis for high-throughput genomics. BMC Genomics 14:397. 10.1186/1471-2164-14-397 23758618 PMC3691752

[B18] Guillen Y. Noguera-Julian M. Rivera J. Casadella M. Zevin A. S. Rocafort M.. (2019). Low nadir CD4+ T-cell counts predict gut dysbiosis in HIV-1 infection. Mucosal Immunol. 12, 232–246. 10.1038/s41385-018-0083-7 30171206

[B19] Hamady Z. Z. Scott N. Farrar M. D. Lodge J. P. Holland K. T. Whitehead T.. (2010). Xylan-regulated delivery of human keratinocyte growth factor-2 to the inflamed colon by the human anaerobic commensal bacterium *Bacteroides ovatus*. Gut 59, 461–469. 10.1136/gut.2008.176131 19736360

[B20] Hamady Z. Z. Scott N. Farrar M. D. Wadhwa M. Dilger P. Whitehead T. R.. (2011). Treatment of colitis with a commensal gut bacterium engineered to secrete human TGF-beta1 under the control of dietary xylan 1. Inflamm. Bowel Dis. 17, 1925–1935. 10.1002/ibd.21565 21830271

[B21] Hensley-McBain T. Wu M. C. Manuzak J. A. Cheu R. K. Gustin A. Driscoll C. B.. (2019). Increased mucosal neutrophil survival is associated with altered microbiota in HIV infection. PLoS Pathog. 15:e1007672. 10.1371/journal.ppat.1007672 30973942 PMC6459500

[B22] Hirao L. A. Grishina I. Bourry O. Hu W. K. Somrit M. Sankaran-Walters S.. (2014). Early mucosal sensing of SIV infection by paneth cells induces IL-1beta production and initiates gut epithelial disruption. PLoS Pathog. 10:e1004311. 10.1371/journal.ppat.1004311 25166758 PMC4148401

[B23] Kaur U. S. Shet A. Rajnala N. Gopalan B. P. Moar P. Himanshu D. (2018). High abundance of genus *Prevotella* in the gut of perinatally HIV-infected children is associated with IP-10 levels despite therapy. Sci. Rep. 8:17679. 10.1038/s41598-018-35877-4 30518941 PMC6281660

[B24] Kehrmann J. Menzel J. Saeedghalati M. Obeid R. Schulze C. Holzendorf V.. (2019). Gut microbiota in human immunodeficiency virus-infected individuals linked to coronary heart disease. J. Infect. Dis. 219, 497–508. 10.1093/infdis/jiy524 30202890

[B25] Kelley C. F. Kraft C. S. de Man T. J. Duphare C. Lee H. W. Yang J.. (2017). The rectal mucosa and condomless receptive anal intercourse in HIV-negative MSM: implications for HIV transmission and prevention. Mucosal Immunol. 10, 996–1007. 10.1038/mi.2016.97 27848950 PMC5433931

[B26] Langille M. G. Zaneveld J. Caporaso J. G. McDonald D. Knights D. Reyes J. A.. (2013). Predictive functional profiling of microbial communities using 16S rRNA marker gene sequences. Nat. Biotechnol. 31, 814–821. 10.1038/nbt.2676 23975157 PMC3819121

[B27] Lee S. C. Chua L. L. Yap S. H. Khang T. F. Leng C. Y. Raja Azwa R. I.. (2018). Enrichment of gut-derived Fusobacterium is associated with suboptimal immune recovery in HIV-infected individuals. Sci. Rep. 8:14277. 10.1038/s41598-018-32585-x 30250162 PMC6155144

[B28] Li S. X. Sen S. Schneider J. M. Xiong K. N. Nusbacher N. M. Moreno-Huizar N.. (2019). Gut microbiota from high-risk men who have sex with men drive immune activation in gnotobiotic mice and *in vitro* HIV infection. PLoS Pathog. 15:e1007611. 10.1371/journal.ppat.1007611 30947289 PMC6448819

[B29] Ling Z. Jin C. Xie T. Cheng Y. Li L. Wu N. (2016). Alterations in the fecal microbiota of patients with HIV-1 infection: an observational study in a Chinese population. Sci. Rep. 6:30673. 10.1038/srep30673 27477587 PMC4967929

[B30] Lozupone C. A. Li M. Campbell T. B. Flores S. C. Linderman D. Gebert M. J.. (2013). Alterations in the gut microbiota associated with HIV-1 infection. Cell Host Microbe 14, 329–339. 10.1016/j.chom.2013.08.006 24034618 PMC3864811

[B31] Lozupone C. A. Rhodes M. E. Neff C. P. Fontenot A. P. Campbell T. B. Palmer B. E. (2014). HIV-induced alteration in gut microbiota: driving factors, consequences, and effects of antiretroviral therapy. Gut Microbes 5, 562–570. 10.4161/gmic.32132 25078714

[B32] Magoc T. Salzberg S. L. (2011). FLASH: fast length adjustment of short reads to improve genome assemblies. Bioinformatics 27, 2957–2963. 10.1093/bioinformatics/btr507 21903629 PMC3198573

[B33] McHardy I. H. Li X. Tong M. Ruegger P. Jacobs J. Borneman J.. (2013). HIV Infection is associated with compositional and functional shifts in the rectal mucosal microbiota. Microbiome 1:26. 10.1186/2049-2618-1-26 24451087 PMC3971626

[B34] Mee M. T. Collins J. J. Church G. M. Wang H. H. (2014). Syntrophic exchange in synthetic microbial communities. Proc. Natl. Acad. Sci. U.S.A. 111, 2149–2156. 10.1073/pnas.1405641111 24778240 PMC4034247

[B35] Mee M. T. Wang H. H. (2012). Engineering ecosystems and synthetic ecologies. Mol. Biosyst. 8, 2470–2483. 10.1039/c2mb25133g 22722235 PMC3430802

[B36] Mehandru S. Poles M. A. Tenner-Racz K. Horowitz A. Hurley A. Hogan C.. (2004). Primary HIV-1 infection is associated with preferential depletion of CD4+ T lymphocytes from effector sites in the gastrointestinal tract. J. Exp. Med. 200, 761–770. 10.1084/jem.20041196 15365095 PMC2211967

[B37] Mutlu E. A. Keshavarzian A. Losurdo J. Swanson G. Siewe B. Forsyth C.. (2014). A compositional look at the human gastrointestinal microbiome and immune activation parameters in HIV infected subjects. PLoS Pathog. 10:e1003829. 10.1371/journal.ppat.1003829 24586144 PMC3930561

[B38] Neff C. P. Krueger O. Xiong K. Arif S. Nusbacher N. Schneider J. M.. (2018). Fecal microbiota composition drives immune activation in HIV-infected individuals. EBioMedicine 30, 192–202. 10.1016/j.ebiom.2018.03.024 29650491 PMC5952409

[B39] Noguera-Julian M. Rocafort M. Guillén Y. Rivera J. Casadellà M. Nowak P.. (2016). Gut microbiota linked to sexual preference and HIV infection. EBioMedicine 5, 135–146. 10.1016/j.ebiom.2016.01.032 27077120 PMC4816837

[B40] Nowak P. Troseid M. Avershina E. Barqasho B. Neogi U. Holm K.. (2015). Gut microbiota diversity predicts immune status in HIV-1 infection. AIDS 29, 2409–2418. 10.1097/QAD.0000000000000869 26355675

[B41] Nowak R. G. Bentzen S. M. Ravel J. Crowell T. A. Dauda W. Ma B.. (2017). Rectal microbiota among HIV-uninfected, untreated HIV, and treated HIV-infected in Nigeria. AIDS 31, 857–862. 10.1097/QAD.0000000000001409 28118207 PMC5342931

[B42] Pinto-Cardoso S. Lozupone C. Briceno O. Alva-Hernandez S. Tellez N. Adriana A.. (2017). Fecal Bacterial Communities in treated HIV infected individuals on two antiretroviral regimens. Sci. Rep. 7:43741. 10.1038/srep43741 28262770 PMC5338340

[B43] Rakoff-Nahoum S. Foster K. R. Comstock L. E. (2016). The evolution of cooperation within the gut microbiota. Nature 533, 255–259. 10.1038/nature17626 27111508 PMC4978124

[B44] Rideout J. R. He Y. Navas-Molina J. A. Walters W. A. Ursell L. K. Gibbons S. M.. (2014). Subsampled open-reference clustering creates consistent, comprehensive OTU definitions and scales to billions of sequences. PeerJ. 2:e545. 10.7717/peerj.545 25177538 PMC4145071

[B45] Serrano-Villar S. Vazquez-Castellanos J. F. Vallejo A. Latorre A. Sainz T. Ferrando-Martinez S.. (2017). The effects of prebiotics on microbial dysbiosis, butyrate production and immunity in HIV-infected subjects. Mucosal Immunol. 10, 1279–1293. 10.1038/mi.2016.122 28000678

[B46] Sun Y. Ma Y. Lin P. Tang Y. W. Yang L. Shen Y.. (2016). Fecal bacterial microbiome diversity in chronic HIV-infected patients in China. Emerg. Microbes Infect. 5:e31. 10.1038/emi.2016.25 27048741 PMC4855070

[B47] Tan H. Zhai Q. Chen W. (2019). Investigations of Bacteroides spp. towards next-generation probiotics. Food Res. Int. 116, 637–644. 10.1016/j.foodres.2018.08.088 30716990

[B48] Tuddenham S. A. Koay W. L. A. Zhao N. White J. R. Ghanem K. G. Sears C. L. (2020). The impact of human immunodeficiency virus infection on gut microbiota alpha-diversity: an individual-level meta-analysis. Clin. Infect. Dis. 70, 615–627. 10.1093/cid/ciz258 30921452 PMC7319268

[B49] Vázquez-Castellanos J. F. Serrano-Villar S. Jimenez-Hernandez N. Soto Del Rio M. D. Gayo S. Rojo D.. (2018). Interplay between gut microbiota metabolism and inflammation in HIV infection. ISME J. 12, 1964–1976. 10.1038/s41396-018-0151-8 29789624 PMC6052150

[B50] Vázquez-Castellanos J. F. Serranovillar S. Latorre A. Artacho A. Ferrús M. L. Madrid N.. (2015). Altered metabolism of gut microbiota contributes to chronic immune activation in HIV-infected individuals. Mucosal Immunol. 8, 760–772 10.1038/mi.2014.107 25407519

[B51] Vesterbacka J. Rivera J. Noyan K. Parera M. Neogi U. Calle M.. (2017). Richer gut microbiota with distinct metabolic profile in HIV infected Elite Controllers. Sci. Rep. 7:6269. 10.1038/s41598-017-06675-1 28740260 PMC5524949

[B52] Villanueva-Millan M. J. Perez-Matute P. Recio-Fernandez E. Lezana Rosales J. M. Oteo J. A. (2017). Differential effects of antiretrovirals on microbial translocation and gut microbiota composition of HIV-infected patients. J. Int. AIDS Soc. 20:21526. 10.7448/IAS.20.1.21526 28362071 PMC5467634

[B53] Vujkovic-Cvijin I. Dunham R. M. Iwai S. Maher M. C. Albright R. G. Broadhurst M. J.. (2013). Dysbiosis of the gut microbiota is associated with HIV disease progression and tryptophan catabolism. Sci. Transl. Med. 5:193ra91. 10.1126/scitranslmed.3006438 23843452 PMC4094294

[B54] Yang L. Poles M. A. Fisch G. S. Ma Y. Nossa C. Phelan J. A.. (2016). HIV-induced immunosuppression is associated with colonization of the proximal gut by environmental bacteria. AIDS 30, 19–29. 10.1097/QAD.0000000000000935 26731752 PMC4813506

[B55] Yu G. Fadrosh D. Ma B. Ravel J. Goedert J. J. (2014). Anal microbiota profiles in HIV-positive and HIV-negative MSM. AIDS 28, 753–760. 10.1097/QAD.0000000000000154 24335481

