# Supplementary material for: Gut Microbiome Changes Associated With HIV Infection and Sexual Orientation
Source: Front Cell Infect Microbiol. 2020 Sep 24;10:434. doi: 10.3389/fcimb.2020.00434 (PMC7546801; doi:10.3389/fcimb.2020.00434)
Supplement: Supplementary file 1 [file Data_Sheet_1.docx]

**Supplementary materials**

**Supplementary Table**

**Supplementary Table 1 Unfiltered sequencing quality results**

| **References** | **Total read counts** | **Average counts per sample** | **Maximum counts per sample** | **Minimum counts per sample** |
| --- | --- | --- | --- | --- |
| **Dillon et al. (2014)** | 3975384 | 124231 | 244804 | 31971 |
| **Lozupone et al. (2014)** | 1147501 | 22067 | 70344 | 3 |
| **Dinh et al. (2015)** | 126105 | 3503 | 18170 | 410 |
| **Vázquez-Castellanos et al. (2015)** | 145552 | 6931 | 16750 | 1103 |
| **Dubourg et al. (2016)** | 7917541 | 74694 | 694269 | 5 |
| **Noguera-Julian et al. (2016)** | 29018173 | 120909 | 1288070 | 53 |
| **Vesterbacka et al. (2017)** | 3436661 | 55430 | 162182 | 5187 |
| **Armstrong et al. (2018)** | 8765197 | 40393 | 129150 | 10702 |
| **Cook et al. (2019)** | 23515495 | 61398 | 154197 | 365 |
| **Lee et al. (2018)** | 2680972 | 58282 | 317482 | 957 |
| **Neff et al. (2018)** | 1984133 | 44092 | 70900 | 17739 |
| **Li et al. (2019)** | 2161195 | 45025 | 138111 | 17721 |

**Supplementary Table 2 Prediction accuracy of different PICRUSt studies related to HIV**

| **References** | **Mean** | **SD** |
| --- | --- | --- |
| **Dillon et al. (2014)** | 0.070 | 0.027 |
| **Lozupone et al. (2014)** | 0.071 | 0.023 |
| **Dinh et al. (2015)** | 0.061 | 0.018 |
| **Vázquez-Castellanos et al. (2015)** | 0.114 | 0.021 |
| **Dubourg et al. (2016)** | 0.070 | 0.035 |
| **Noguera-Julian et al. (2016)** | 0.099 | 0.031 |
| **Vesterbacka et al. (2017)** | 0.092 | 0.027 |
| **Armstrong et al. (2018)** | 0.076 | 0.019 |
| **Cook et al. (2019)** | 0.072 | 0.020 |
| **Lee et al. (2018)** | 0.049 | 0.014 |
| **Neff et al. (2018)** | 0.077 | 0.018 |
| **Li et al. (2019)** | 0.079 | 0.018 |

**Supplementary Table 3 Prediction accuracy of different PICRUSt studies related to MSM**

| **References** | **Mean** | **SD** |
| --- | --- | --- |
| **Dinh et al. (2015)** | 0.051 | 0.019 |
| **Noguera-Julian et al. (2016)** | 0.100 | 0.032 |
| **Vesterbacka et al. (2017)** | 0.092 | 0.027 |
| **Armstrong et al. (2018)** | 0.076 | 0.019 |
| **Lee et al. (2018)** | 0.049 | 0.014 |
| **Li et al. (2019)** | 0.079 | 0.018 |

**Supplementary Figure**

**Figure Legends**

Supplementary Figure 1. Forest plots of twelve studies, comparing HIV+ to HIV- individuals.

Supplementary Figure 2. Forest plots restricted to man, comparing HIV+ to HIV- individuals.

Supplementary Figure 3. Forest plots restricted to woman, comparing HIV+ to HIV- individuals.

Supplementary Figure 4. Forest plots restricted to MSM, comparing HIV+ to HIV- individuals.

Supplementary Figure 5. Forest plots restricted to non-MSM, comparing HIV+ to HIV- individuals.

Supplementary Figure 6. Forest plots restricted to age < 45, comparing HIV+ to HIV- individuals.

Supplementary Figure7. Forest plots restricted to age ≥ 45, comparing HIV+ to HIV- individuals.

Supplementary Figure 8. Forest plots restricted to BMI = 18.5-23.9, comparing HIV+ to HIV- individuals.

Supplementary Figure 9. Forest plots restricted to BMI = 24-27.9, comparing HIV+ to HIV- individuals.

Supplementary Figure 10. Forest plots restricted to BMI > 28, comparing HIV+ to HIV- individuals.

Supplementary Figure 11. Forest plots restricted to CD4^+^T cell count < 500, comparing HIV+ to HIV- individuals.

Supplementary Figure 12. Forest plots restricted to CD4^+^T cell count ≥ 500, comparing HIV+ to HIV- individuals.

Supplementary Figure 13. Forest plots restricted to non-ART, comparing HIV+ to HIV- individuals.

Supplementary Figure 14. Forest plots restricted to ART, comparing HIV+ to HIV- individuals.

Supplementary Figure 15. Forest plots restricted to HIV Viral load < 200, comparing HIV+ to HIV- individuals.

Supplementary Figure 16. Forest plots restricted to HIV Viral load ≥ 200, comparing HIV+ to HIV- individuals.

Supplementary Figure 17. Forest plots of six studies, comparing MSM to non-MSM.

Supplementary Figure 18. Forest plots restricted to HIV+, comparing MSM to non-MSM.

Supplementary Figure 19. Forest plots restricted to HIV-, comparing MSM to non-MSM.

Supplementary Figure 20. Forest plots restricted to age < 45, comparing MSM to non-MSM.

Supplementary Figure 21. Forest plots restricted to age ≥ 45, comparing MSM to non-MSM.

Supplementary Figure 22. Forest plots restricted to BMI = 18.5-23.9, comparing MSM to non-MSM.

Supplementary Figure 23. Forest plots restricted to BMI = 24-27.9, comparing MSM to non-MSM.

Supplementary Figure 24. Forest plots restricted to BMI > 28, comparing MSM to non-MSM.

**Supplementary Figure 1**

**Supplementary Figure 2**

**Supplementary Figure 3**

**Supplementary Figure 4**

**Supplementary Figure 5**

**Supplementary Figure 6**

**Supplementary Figure 7**

**Supplementary Figure 8**

**Supplementary Figure 9**

**Supplementary Figure 10**

**Supplementary Figure 11**

**Supplementary Figure 12**

**Supplementary Figure 13**

**Supplementary Figure 14**

**Supplementary Figure 15**

**Supplementary Figure 16**

**Supplementary Figure 17**

**Supplementary Figure 18**

**Supplementary Figure 19**

**Supplementary Figure 20**

**Supplementary Figure 21**

**Supplementary Figure 22**

**Supplementary Figure 23**

**Supplementary Figure 24**
